# Leptospirosis and characterization of *Leptospira* isolates from patients in Koprivnica-Križevci County, Croatia from 2000–2004

**DOI:** 10.1099/acmi.0.000431

**Published:** 2023-04-26

**Authors:** Ljiljana Mišić-Majerus, Tjaša Cerar Kišek, Eva Ružić-Sabljić

**Affiliations:** ^1^​ Department of Infectious Diseases, General Hospital "Dr. Tomislav Bardek", Željka Selingera 1, Koprivnica, Croatia; ^2^​ Faculty of Medicine, Institute of Microbiology and Immunology, University of Ljubljana, Zaloška 4, Ljubljana, Slovenia

**Keywords:** Leptospirosis, *Leptospira *isolation, *Leptospira *species, *Leptospira *serogroup/serovar, MAT, Koprivnica-Križevci County, Croatia, MLST, Sequence type

## Abstract

**Introduction.:**

Leptospirosis, caused by spirochetes of the genus *Leptospira *, is present in the Koprivnica-Križevci County area, Croatia. Clinical manifestation can range from asymptomatic, short-term mild, non-specific febrile disease, to severe forms with high mortality rates.

**Aim.:**

The aim of the study was to valuate culture in front of microscopic agglutination test (MAT) for diagnosis of infection, and to evaluate clinical and laboratory features of the disease. Moreover, we want to characterize *Leptospira* strains involved in infection in Koprivnica-Križevci County, Croatia.

**Methods.:**

We included 68 patients with clinical presentation consistent with leptospirosis collected in a 5-year period (2000–2004). Clinical samples (blood, urine and cerebrospinal fluid, CSF) were inoculated in Kolthoff’s medium; species of isolated *Leptospira* strains was determined with Tm of real-time PCR, serogroup/serovar with MAT and NotI-RFLP analysis. Demonstration of specific antibodies in patients’ sera was done using microscopic agglutination test.

**Results.:**

*
Leptospira* was isolated from the blood of 14/51(27.5 %) patients and the most often identified serogroup/serovar was Icterohaemorrhagiae (8/10, 80%) followed by Grippotyphosa (10%). Regarding to species level, 8/10 isolated belonged to *L. interrogans sensu stricto* and one to *L. kirschneri* species. MAT was carried out on 51 patients with suspected leptospirosis, and was positive in 11/51(21.5 %) patients. Most of our patients presented with moderate severe symptoms, were hospitalized from August to October, and were infected mainly during the work or recreation in our county. The frequency of particular clinical features and pathological laboratory findings correlated with the severity of the clinical condition.

**Conclusions.:**

Leptospirosis can be confirmed microbiologically, culture and MAT contributed almost equally to the diagnosis of infection. Serovar Icterohaemorrhagiae was found as the dominant one, and *L. interrogans sensu stricto* as dominant species in our county. Epidemiological data shown that leptospirosis occurs seasonally, affects the rural population, and most commonly is presented with moderate severe clinical course.

## Data Summary

The authors confirm all supporting data, code and protocols have been provided within the article or through supplementary data files.

## Introduction

Leptospirosis is zoonosis present all over the world, especially in tropical and subtropical areas. The pathogenic spirochetes from the genus *Leptospira* spp., generally named *Leptospira interrogans sensu lato* (*sensu lato* – in broad sense) represent etiological agent of the infection. It is the most frequent zoonotic disease [1, 2]. Significant part of free living and domestic animals are infected with *Leptospira *, and for long-term excrete spirochetes by urine. Humans are infected by direct contact with the body fluids of an infected animal or indirectly from the ground, or water contaminated with the urine of infected animals [1, 2]. *Leptospira* enter the human body most often through damaged skin or exposed mucous membranes (including eye and pharynges). In the last years, reported infections were associated with recreational activities in the nature, with passengers and/or soldiers going to tropical and subtropical areas [2–5]. Clinical manifestation of leptospirosis ranges from asymptomatic, light short-term, ‘flu-like’ febrile illnesses, to severe forms with high mortality rates [6–9]. Direct and indirect microbiological methods are used to confirm the infection [10–12]. For severe clinical disease, specific antimicrobial treatment as early as possible is recommended that will alleviate the acute symptoms of the disease, shorten the course of infection, and prevent excretion of *Leptospira* with urine. Preventive measures are difficult to implement [2, 13].

The county of Koprivnica-Križevci is located in the north-western part of Croatia (https://kckzz.hr/poziv-na-javni-uvid-i-raspravu-prijedlog-proglasenja-regionalnog-parka-mura-drava-2/). About 60–70 % of the area is cultivable plains and valleys, while the rest is hilly. Rural people here are intensely involved in raising livestock (especially pigs), and wastewater often runoff into nearby streams. The presence of small mammals (mice, rats) on the environment of rural households and the creeks is a daily occurrence. Leptospirosis was already reported in this region in year 1977 [14].

The aim of the study was to identify *Leptospira* strains involved in infection in Koprivnica-Križevci County, Croatia. Moreover, we wished to value culture in front of MAT for diagnosis of infection, and to link clinical and laboratory features with the severity of disease.

## Methods

In the present study we included 68 patients with clinical manifestations that were suggestive of leptospirosis during the period 2000–2004. The patients were admitted to the Department of Infectious Diseases, ‘Dr. Tomislav Bardek’ General Hospital in Koprivnica-Križevci County, Croatia (https://kckzz.hr/poziv-na-javni-uvid-i-raspravu-prijedlog-proglasenja-regionalnog-parka-mura-drava-2/). In addition to the clinical and laboratory examination, collected anamnestic and epidemiological data, microbiological tests (cultivation and serology) were performed, and isolated leptospira strains were analysed to species level and serogroup/serovar.

### Patients and clinical manifestations

In the study we included patients with clinical suspicion of leptospirosis who were examined and treated in the Department of Infectious Diseases of the General Hospital ‘Dr. Tomislav Bardek’ in Koprivnica during the period 2000–2004. To evaluate the severity of the disease, we used a modified Croatian score scale for assessing the severity of the disease in patients with haemorrhagic fever with kidney syndrome reported by Kuzman [15]. Based on clinical features, patients were divided into four groups: mild, moderate, severe and extremely severe. All other information about the patient were collected from the patient’s history of the disease. Some patients were also included into the epidemiological follow-up study over three time periods managed by another institution as described previously [16].

### Patients and clinical specimens

The study included 68 patients. All patients were examined on the day of their arrival to the hospital and onwards.

The first clinical samples for microbiological testing were sent to laboratory in various combinations: (1) for *Leptospira* cultivation only we received blood from 23 patients, urine from three patients, blood and urine simultaneously from 11 patients, and blood, urine and cerebrospinal fluid (CSF) simultaneously from one patient; (2) for detecting specific antibodies only with microscopic agglutination test (MAT) we received blood from 17 patients; (3) combination of *Leptospira* culture and MAT were received as follows: blood for MAT and culture from 11 patients; blood for MAT and blood and urine for culture from two patients.

Thus at the first clinical examination, we performed cultivation on a total of 48 blood samples, 16 urine samples and one CSF sample; serological test MAT on 30 blood samples.

We retested 29 patients in the interval of 7 days to 6 months, some were tested for antibodies only, some for culture only, and some for both. Taking together, culture was performed for 23 patients (20 from urine and three from blood samples) while MAT test was performed for 21 patients.

### Cultivation of clinical specimens

Blood, urine and CSF were taken from patients the first day of hospitalization that was, regarding to anamnestic data the first or second week of acute illness. Samples were immediately inoculated into tubes filled with Korthoff’s medium for *Leptospira* cultivating [12, 17] and sent to the laboratory by courier. For that purpose, clinicians were supported with tubes containing 5 ml of Korthoff’s medium, and inoculated up to 5 ml of particular sample. In the laboratory, tubes were re-inoculated into three to four tubes with fresh medium (1 to 2 ml per tube), samples were incubated at 28 °C, and weekly checked for the presence of spirochetes. Samples were declared as negative if no growth was found after 9 weeks of incubation [12, 17].

### Identification of isolated *Leptospira* strains

For particular isolated *Leptospira* strain we determined serogroup, serovar, species and sequence type (ST).

#### Serogroup determination

To determine the serogroup status of the isolated *Leptospira* strain, strains were incubated with rabbit antisera representing some recognized serogroups at dilution 1 : 400 and more; reference sera were obtained from the Pasteur Institute, Paris and the Royal Institute for Tropical Medicine, Amsterdam. This included antisera for serogroups: Grippotyphosa, Sejroe, Australis, Pomona, Canicola, Icterohaemorrhagiae, Tarassovi, Saxkoebing, Ballum, Batavia, Poi, Hardy, Autumnalis and Mitis. After 45 min of incubation at 37 °C, samples were checked in dark field microscopy and determined the highest agglutination titre for each antiserum. The unknown *Leptospira* strain was placed in serogroup regarding to the highest titre agglutination [12].

#### Serovar determination

Serovars of isolated *Leptospira* strains were determined by NotI restriction fragment length polymorphism (NotI-RFLP). For that purpose, tubes with growing culture were centrifuged, sediment washed with buffer, and approximately 10^8^ cells ml^−1^ were mixed with agarose and put into moulds. Whole agarose blocks were lysed in lysis buffer containing lysozyme (1 mg ml^−1^) and RNase (10 µg ml^−1^) and digested in digestion buffer containing proteinase K (0.5 mg ml^−1^). After exhaustive block washing, DNA was restricted with NotI (20 E/200 µl) and performed to PFGE. Restriction patterns were separated for 24 h with ramping pulse times of 5 to 70 s; molecular size markers of 50 to 1000 kb (Sigma) were included in each electrophoresis. Restriction sample of isolated *Leptospira* strain was compared with the restriction sample of the reference strain of a known serovar [17, 18].

#### Species identification

The *Leptospira* species was identified by a slightly modified method developed by Merien *et al*. [19]. We used commercial mastermix LC FastStart DNA Master SYBR Green I mastermix, from Roche, Germany. Identification is based on melting temperature (Tm) determination of the PCR product of the gene LAO322.

#### Multilocus sequence typing (MLST) analysis

MLST was performed based on two published scheme targeting seven (MLST scheme 2: adk_2, glmU_2, icdA_2, lipL32_2, lipL41_2, mreA_2, pntA_2) and six loci (MLST scheme 3: adk_3, icdA_3, lipL32_3, lipL41_3, rrs2_3, secY_3) [20, 21] and analysed by using Multilocus Sequence Typing Module in CLC Main Workbench 7 (Qiagen, Germany). We constructed a minimum spanning tree by using BioNumerics version 7.6 software (Applied Maths, Austin, TX, USA) and compared *Leptospira* sequence type (ST). Comparison of strains from different regions included MLSTs of all *Leptospira* isolates in the *Leptospira* MLST database as of 2019-07-09 (https://pubmlst.org/bigsdb?db=pubmlst_leptospira_isolates).

### Serological testing

Serum samples were taken in the acute phases of the disease, recovalescent samples in 4 to 6 months after first testing. Samples were further processed in Laboratory for Diagnosis of Borreliosis and Leptospirosis at Institute for Microbiology and Immunology of the Faculty of Medicine in Ljubljana. For antibody determination, MAT was performed used [12, 17]. The test includes 14 *L *. interrogans* sensu lato* serovars present in our geographic area: Grippotyphosa, Sejroe, Australis, Pomona, Canicola, Icterohaemorrhagiae, Tarassovi, Saxkoebing, Ballum, Batavia, Poi, Hardy, Autumnalis and Mitis. In patients receiving only one sample, titre 1 : 100 was taken as a significant according to the World Health Organization’s recommendation, and in patients receiving several sera, only those with a fourfold increase in antibody titre (or seroconversion), both according to corresponding clinical findings [12, 17]. Serological testing of some of included patients were also performed in another laboratory and were included in epidemiological study reported previously [16].

### Statistical analysis

Data were summarized with frequencies and percentages. McNemar's test with the level of significance set at *P*<0.05 was used for statistical comparison of data.

## Results

In the analysis, 68 patients with clinical signs suspected to leptospirosis were included. All patients were screened at the day of arrival to the hospital, 29 of them (42.6 %) were followed up and retested for antibodies. Leptospirosis was confirmed by culture and/or by serology (MAT); isolated *Leptospira* strains were identified to species, serovar and serogroup level. Only for patients with microbiologically confirmed infection (positive culture and/or MAT), clinical and laboratory data were reported. Incidence rate, epidemiology and clinical course of infection for part of these patients were reported previously [16].

### Sample cultivation and *Leptospira* spp. isolation

Cultivation was performed on 65 clinical specimens (48 blood, 16 urine and one cerebrospinal fluid) of 51 patients at their first examination; for the remaining 17 patients, cultures were not sent to the laboratory. *Leptospira* was isolated from the blood of 14/51(27.5 %) patients, in all of them, blood was taken for cultivation the same day as they entered the hospital. All other clinical samples remained culture negative. Growing *Leptospira* strains were subcultured and stored at liquid nitrogen until further analysis.

Among the isolated *Leptospira* strains there were four strains that failed to grow up after been stored at liquid nitrogen or grew slightly, two of them also were contaminated, and we could not perform further strain identification while the rest of the ten strains we managed to identify. Patients whom *Leptospira* was isolated from the blood and identified as well as patients’ clinical features are shown in Table 1.

**Table 1.** Data about patients whom *Leptospira* was isolated from the blood according to the severity of the clinical course, *Leptospira* serogroup (agglutination titre), serovar (NotI-restriction fragment length polymorphism, RFLP), species (PCR based Tm) determination and MLST analysis (scheme 2 and 3 reported as sequence type, ST)

No.	Patient	Clinical course	Sample	Determined *Leptospira* serogroup (titre)	Determined *Leptospira* serovar (NotI-RFLP)	Determined *Leptospira* species	MLST scheme 2 (ST)	MLST scheme 3 (ST)
1	Z.D.	Mild	Blood	Grippotyphosa 1 : 800	Grippotyphosa as Moskva V	/*	/*	/*
2	S.M.	Severe	Blood	Icterohaemorrhaiae 1 : 1600	Icterohaemorrhaiae as RGA, Copenhageni (Wijnberg)	*L. interrogans sensu stricto*	/*	/*
3	M.B.	Severe	Blood	Icterohaemorrhaiae 1 : 800	Icterohaemorrhaiae as RGA, Copenhageni (Wijnberg)	*L. interrogans sensu stricto*	*47*	*2*
4	M.S.	Severe	Blood	Icterohaemorrhaiae 1 : 1600	Icterohaemorrhaiae as RGA, Copenhageni (Wijnberg)	*L. interrogans sensu stricto*	*47*	*2*
5	S.Z.	Severe	Blood	Batavia 1 : 6400 Tarassovi 1 : 3200	Special profile, no reference strain for comparing	* L. kirschneri *	150	144†
6	M.I.	Moderate severe	Blood	Icterohaemorrhaiae 1 : 1600	Icterohaemorrhaiae as RGA, Copenhageni (Wijnberg)	*L. interrogans sensu stricto*	*47*	*2*
7	L.K.	Severe	Blood	Icterohaemorrhaiae 1 : 800	Icterohaemorrhaiae as RGA, Copenhageni (Wijnberg)	*L. interrogans sensu stricto*	*47*	*2*
8	K.S.	Moderate severe	Blood	Icterohaemorrhaiae 1 : 3200	Icterohaemorrhaiae as RGA, Copenhageni (Wijnberg)	*L. interrogans sensu stricto*	*47*	*2*
9	S.J.	Severe	Blood	Icterohaemorrhaiae 1 : 400	Icterohaemorrhaiae as RGA, Copenhageni (Wijnberg)	*L. interrogans sensu stricto*	/*	/*
10	M.K.	Moderate severe	Blood	Icterohaemorrhaiae 1 : 800	Icterohaemorrhaiae as RGA, Copenhageni (Wijnberg)	*L. interrogans sensu stricto*	/*	/*

*Unsuccessful subculturing from liquid nitrogen.

†New sequence type (ST).

### *Leptospira* identification

Serogroup and serovar of isolated *Leptospira* were identified for nine of ten well growing strains.

**Fig. 1.**
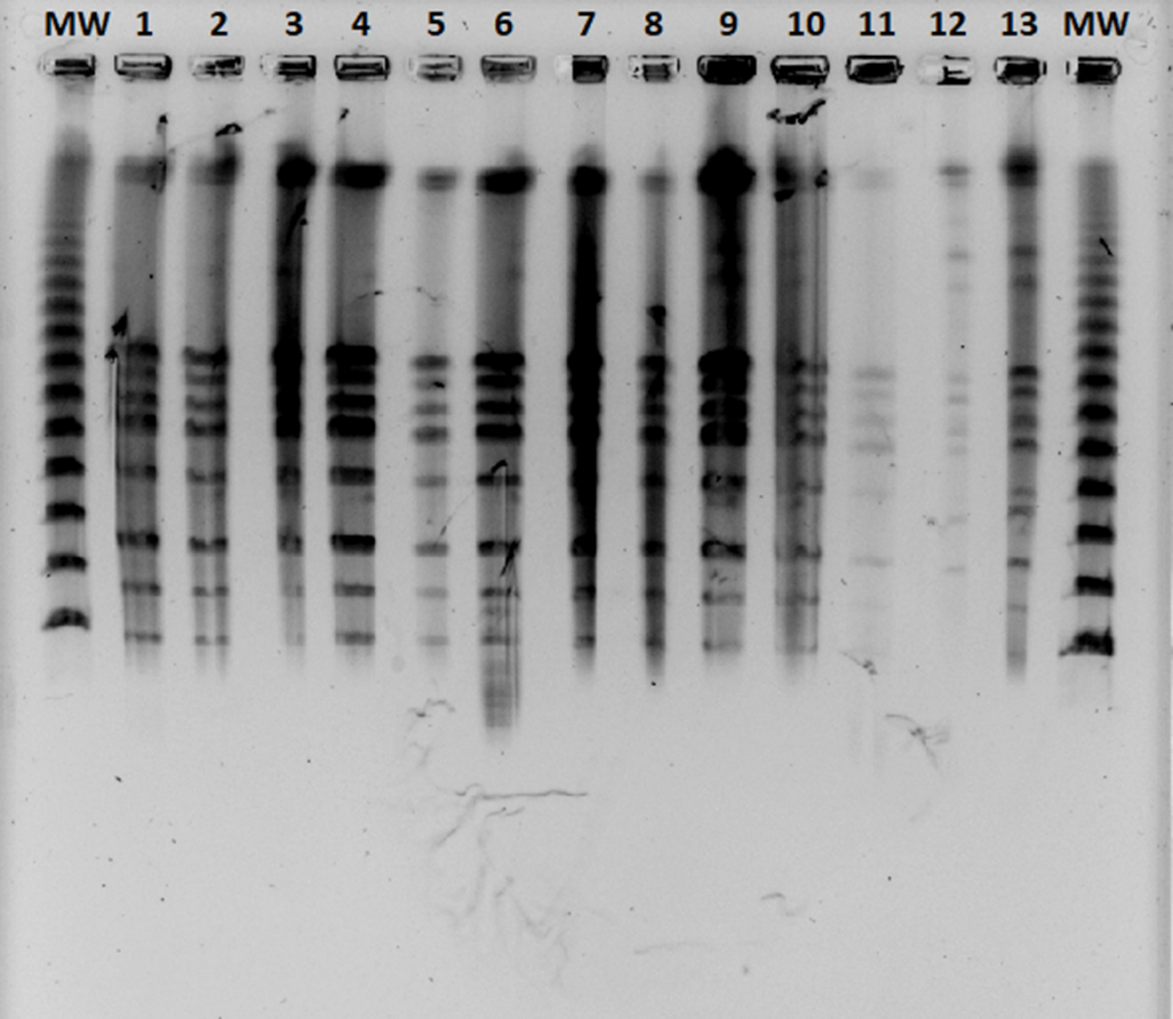
NotI restriction fragment length polymorphism (NotI-RFLP) profile of some clinical and reference *Leptospira* strains. MW – molecular weight standard; lanes 2–10 *Leptospira* strains from Table 1: lane 2 SM, lane 3 MB, lane 4 and 5 MS, lane 6 and 11 MI, lane 7 LK, lane 8 KS, lane 9 SJ and lane 10 MK); lanes 11–13 reference *Leptospira* strains of serogroup Icterohaemorrhagiae (lanes 11 and 13 serovar Copenhageni strain Wijnberg, lane 12 serovar Icterohaemorrhagiae strain RGA).

Regarding serogroup and serovar, most patients, eight of ten (80 %) were infected with *Leptospira* Icterohaemorrhagiae, one patient (10 %) with *Leptospira* Grippotyphosa (Table 1). For strain of patient S.Z. (patient no. 5 in Table 1) we failed to select the serogroup because the strain agglutinated with hyper-immune serum from the Batavia (1 : 6400) and Tarassovi (1 : 3200) serologic group, and we also failed to delineate serovar because the isolated strain had its own NotI-RFLP profile that we did not have among our reference *Leptospira* strains. NotI-RFLP of some clinical and reference strains is present in Fig. 1.

**Fig. 2.**
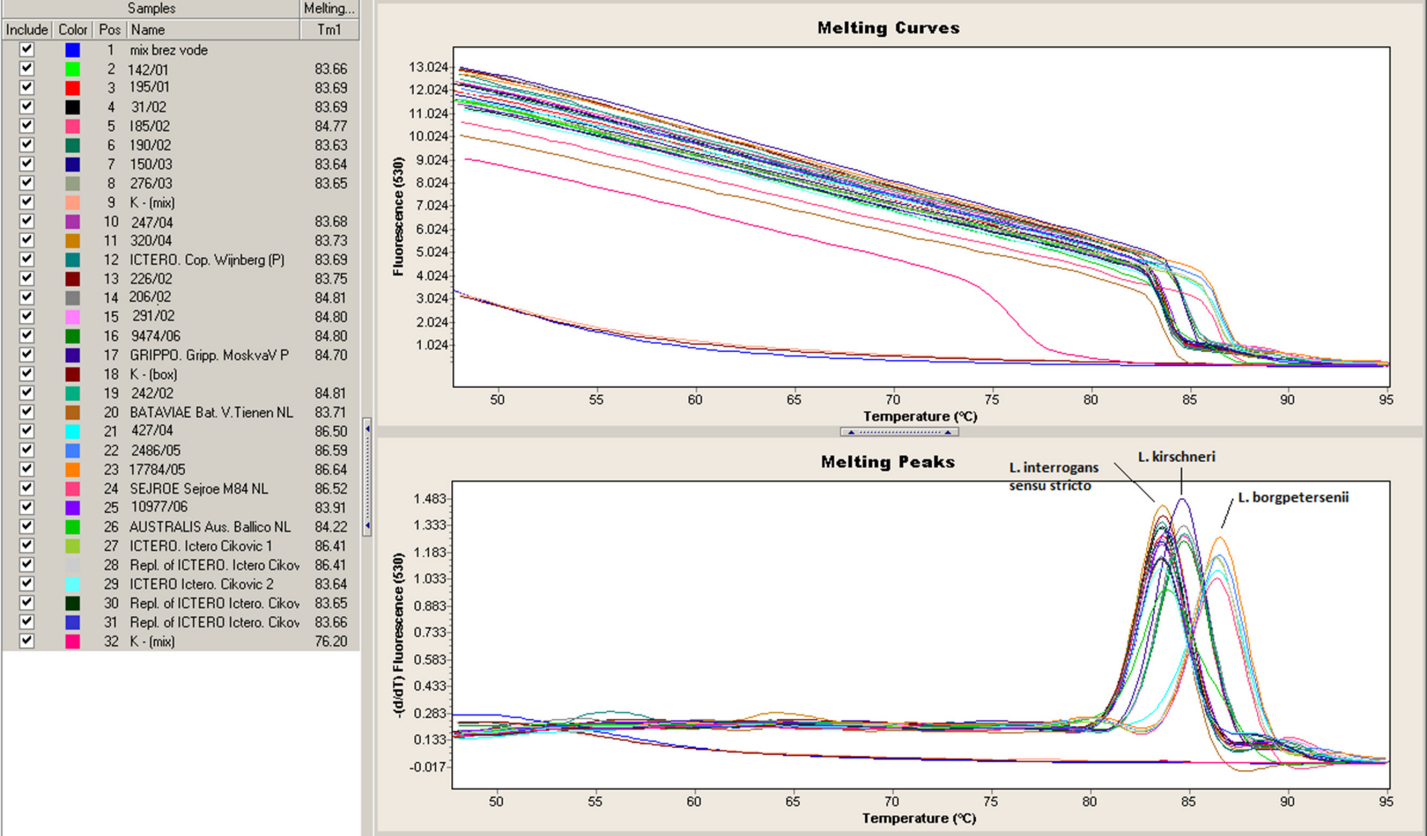
Leptospira species identification by melting temperature (Tm) determination of the PCR product of the gene LAO322, particular Tm peak defines *Leptospira* species; included are clinical and reference strains.

Regarding species identification, we successfully identified nine/ten well growing *Leptospira* strains (Table 1, Fig. 2); for patient Z.D. (no. 1 in Table 1, serovar Grippotyphosa) we could not identified species because we could not reculture the strain from the liquid nitrogen years after it was stored while its serovar was identified at the year of its isolation. Among the isolated strains, eight of ten were identified as *L. interrogans sensu stricto* and one as *L. kirschneri* (Table 1).

**Fig. 3.**
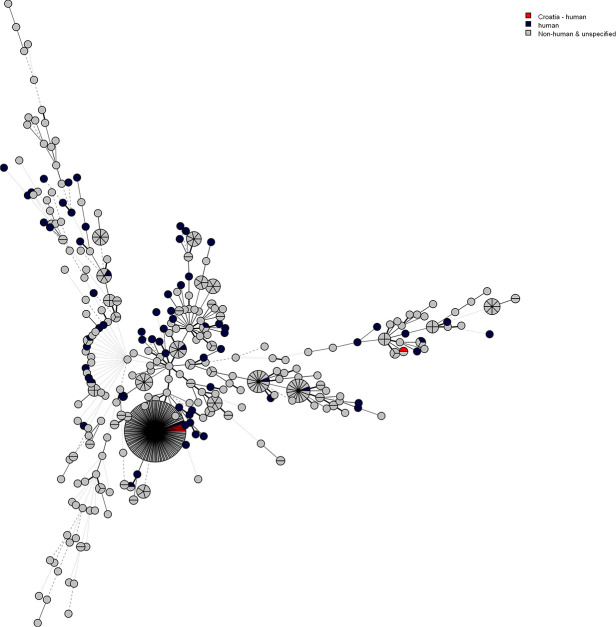
Minimum spanning tree of six *Leptospira* strains (red) isolated in Croatia in comparison to 528 *Leptospira* isolates, available at the *Leptospira* MLST database as of 2019-07-09.

Using MLST we have characterized six isolates that we were able to reculture from liquid nitrogen. We defined different STs: with scheme 2: ST 47 (majority of strains) and ST 150 while with scheme 3: ST 2 (majority of strains) and ST 144; ST 144 was the new one (Table 1 and Fig. 3). Six *Leptospira* strains isolated in Croatia were placed together with 528 *Leptospira* isolates, available at *Leptospira* MLST database scheme 2 and their minimum spanning tree is shown on Fig. 3. ST 144 defined with scheme 3 was the same as ST 150 defined with scheme 2, and lies on the right branch on Fig. 3.

### Microscopic agglutination test

Test MAT was performed for 30 patients the day they entered into the hospital; specific antibodies were detected in 9/30(30 %) patients (Table 2). Among serologically positive patients, there was only one culture positive patient whom *Leptospira* was isolated from the blood (patient S.Z. no. 5 in Table 1).

**Table 2.** Contributions of culture and/or microagglutination test (MAT) to confirm leptospirosis in 24/68(35.3 %) microbiologically confirmed patients. Rest of patients (44/68; 64.7 %) were microbiologically negative

Tests and results	First testing No. of positive patients	Retesting No. of new positive patients	ALL
Culture positive only	11*	0	11
MAT positive only	3	1	4
Culture positive and MAT positive	1	0 (1*)	1 (1*)
Culture positive, MAT negative	2	0	2
Culture negative, MAT positive	5	1	6
**ALL**	**22**	**2**	**24** (25*)

*Culture positive patient (M.B., no. 3 in Table 1) whom MAT was not performed at first examination but in second, positive MAT in second testing did not contribute to a new diagnosed patient.

Subsequently 21 patients was tested with MAT and five were found positive but only two of these MAT positive samples contribute to overall leptospirosis diagnosis because three of them were already culture positive patients (Table 2).

In total, the test MAT carried out on 51 patients with suspected leptospirosis, after the first and repeated testing, confirmed leptospirosis in 11/51(21.5 %) patients.

### Valuation of culture and MAT for confirming leptospirosis

From Table 2 is evident that leptospirosis was confirmed by microbiological tests in 22 patients the first day of their examination. By further testing, two more patients remained positive, both had positive MAT. Generally, culture and MAT contributed almost equally to the diagnosis of leptospirosis; there were 13 patients only culture positive and 10 only MAT positive, only one patient was culture and MAT positive. Culture and MAT complemented each other in confirming leptospirosis.

There was a small group of only 13 patients that cultivation and MAT were taken simultaneously at the first medical examination. Comparing the tests’ results in these patients, we found following combinations: one culture and MAT positive patient, two culture positive/MAT negative patients, six culture negative/MAT positive patients, and four culture and MAT negative patients. Among 7/13 MAT positive patients only one was culture positive, and among 3/13 culture positive patients only one was MAT positive. Valuated results of simultaneously performed test for leptospirosis confirmation, culture contributed to diagnosis in 3/13(23.1 %) patients and MAT in 6/13(46.2 %) patients, but no statically significant difference was found (*P*=0.453).

### Microbiological confirmation of leptospirosis in patients

In the period from 2000 to 2004, 68 patients with clinical suspicion of leptospirosis were examined. Since the clinical samples were sent to the laboratory in various combinations (only for culture, only for serology, both), and time period, leptospirosis was demonstrated by various combinations of tests as shown in Table 2. In total, leptospirosis was microbiologically confirmed in 24/68(35.3 %) patients.

### Epidemiological background of infection

**Fig. 4.**
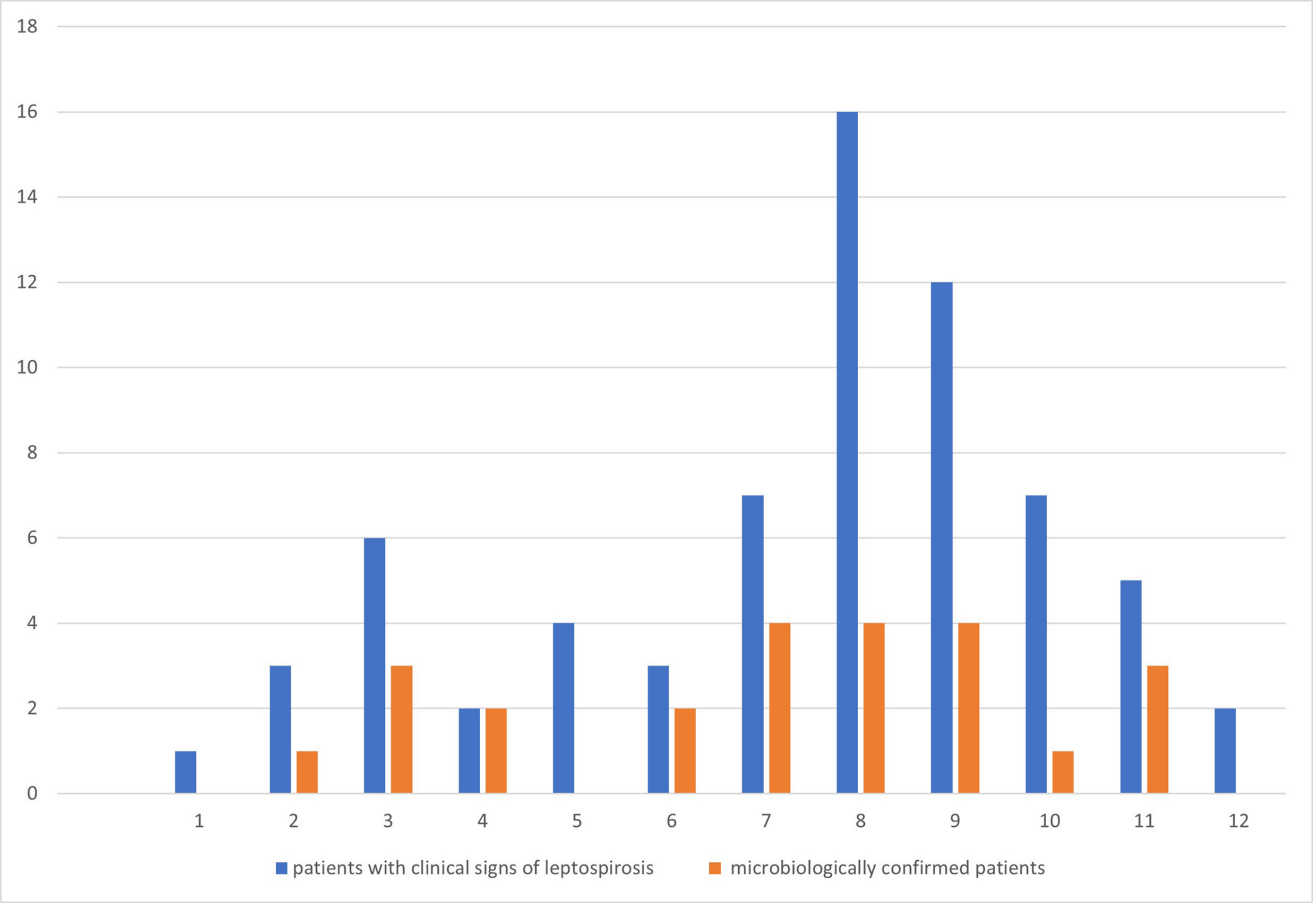
Seasonal distribution of leptospirosis cases (*y*-axis) from January to December (*x*-axis: 1–12) in Koprivnica-Križevci County, Croatia.

According to anamnestic data and clinical presentation, all 68 presented patients were suspected to be infected with *Leptospira *. Fig. 4 shows seasonal distribution of 48/68(70.5 %) patients with clinical signs of leptospirosis that were treated in our hospital, and highlight that most of the patients were treated from August to November. Epidemiological data that were collected retrospectively for microbiologically confirmed patients (but for only 17/24, 70.8 %) indicate that infection can be associated with living, recreating and working in the rural environment, contact with domestic animals and free living small mammals. Among them, three (12.5 %) children have been infected during swimming in rural creek and waterfowls, and four (16.6 %) during fishing.

### Demographic, clinical and laboratory characteristics of patients

During the analysed period, we confirmed leptospirosis either by direct or indirect microbiological methods in 24/68(35.3 %) patients; 3/24(12.5 %) were children under 13 years, and 21/24(87.5 %) adults, 17/24(70, 8 %) male and 7/24(29.1 %) female, aged 13 to 75 (mean age 47 years). Patients entered the hospital between the second and the seventh day of the beginning of their acute clinical signs (mean value 4.3 days).

Regarding to severity of clinical course of infection, patients were grouped according to Kuzman [15]; results are shown in Table 3. Moderate severe (10/24; 41.6 %) and severe (7/24, 29.1 %) clinical manifestation was predominated.

**Table 3.** Distribution of patients (no. 24) with microbiologically confirmed leptospirosis either by culture and/or microagglutination test (MAT) regarding to clinical course of infection

Clinical course of infection	Microbiologically confirmed leptospirosis Culture positive only	Microbiologically confirmed leptospirosis MAT positive only	Microbiologically confirmed leptospirosis Culture positive and MAT positive	ALL
**Mild**	3	1	–	4 (16.7 %)
**Moderate severe**	6	4	–	10 (41.6)
**Severe**	2	4	1	7 (29.2)
**Extreme severe**	2	1	–	3 (12.5)
**Fatal**	–	–	–	–
**ALL**	**13**	**10**	**1**	**24** (100 %)

Because isolation of the causative agent presents the gold standard for confirming infection aetiology, we paid special attention to recording clinical features and laboratory findings for patients with positive culture, 14 met the criteria. The results are shown in Tables 4 and 5. Based on culture confirmed leptospirosis, the frequency of clinical features related to leptospirosis ranged from 57.1–78.6 %. There were 8/14 patients with jaundice and oliguria (to anuria) and 10 and 11/14 patients with marked myalgia and eye injection (Table 4). Regarding to laboratory findings, elevated sedimentation and LDH was observed in all patients with leptospirosis (Table 5) while 10 to 12/14(71.4–85.7 %) patients had pathological values of CRP, platelets, AST, CK, ALT and leukocytes that followed the severity of clinical manifestation (Table 5).

**Table 4.** Frequency of clinical symptoms and signs of leptospirosis in patients with positive culture (no. 14) regarding to severity of clinical course of infection

Clinical symptoms and/or signs	Clinical course of infection/no. of patients with positive culture				
	Extremely sever	Sever	Moderate sever	Mild	All
fever	2	3	6	3	14 (100 %)
chills	2	3	5	2	12 (85.7 %)
eye injection	2	3	5	1	11 (78.6 %)
myalgia	2	2	4	2	10 (71.4 %)
hypotension	2	3	5	0	10 (71.4 %)
weakness	2	3	5	0	10 (71.4 %)
headache	2	3	4	0	9 (64.3 %)
vomiting	2	2	5	0	9 (64.3 %)
jaundice	2	3	3	0	8 (57.1 %)
oliguria, anuria	2	2	4	0	8 (57.1 %)
abdominal pain	2	3	2	1	8 (57.1 %)
diarrhoea	2	2	3	0	7 (50.0 %)
blooding	2	2	2	0	6 (42.9 %)
cough	2	0	0	0	4 (28.6 %)
dizziness	2	1	1	0	4 (28.6 %)
**All**	**2**	**3**	**6**	**3**	**14** (100 %)

**Table 5.** Pathological laboratory findings in patients with culture confirmed leptospirosis (no. 14)

Laboratory findings	No. of patients				
	Extremely sever	Sever	Moderate sever	Mild	All
erythrocyte sedimentation rate, SE (>23 mm/3,6 ks)	2	3	6	3	14 (100 %)
Lactate dehydrogenase, LDH (>241 U l^−1^)	2	3	6	3	14 (100 %)
Thrombocytes (<158 x 10^9^ l^−1^)	2	3	6	1	12 (85.7 %)
C-reactive protein, CRP (>5 mg l^−1^)	2	3	5	2	12 (85.7 %)
aspartate aminotransferase, AST (>38 U l^−1^)	2	3	5	1	11 (78.6 %)
creatinine kinase, CK (>177 U l^−1^)	2	3	5	1	11(78.6 %)
Leukocytosis (˃9,7×10^9^ l^−1^)	1	3	4	2	10(71.4 %)
neutrophils (>2,0×10^9^ l^−1^)	2	3	4	1	10(71.4 %)
alanine aminotransferase, ALT (>48 U l^−1^)	2	3	4	1	10(71.4 %)
Bilirubin (>20 millimol l^−1^)	2	3	3	0	8(57.1 %)
creatinine (>104 millimol l^−1^)	2	3	3	0	8(57.1 %)
Amylases in blood (>220 U l^−1^)	0	1	1	0	2(14.3 %)
Amylases in urine (>1000 U l^−1^)	0	1	1	0	2(14.3 %)
**All**	**2**	**3**	**6**	**3**	**14**(100 %)

## Discussion

Leptospirosis is present in the Koprivnica-Križevci County (https://kckzz.hr/poziv-na-javni-uvid-i-raspravu-prijedlog-proglasenja-regionalnog-parka-mura-drava-2/), the first patients were diagnosed in 1959 [22] while first epidemiological data are from 1977 [14]. Present study covered the period 2000–2004 during which 68 patients were suspected of having leptospirosis. Our main interest was to identify human *Leptospira* strains isolated in our county and to evaluate microbiologic tests for diagnosis of the infection. Comparing data from 1970 to 1975, we found two times lower incidence of leptospirosis and shift in the frequency of the disease to the autumn months (Fig. 4) but still association with the rural population [11, 14, 16]. Baranton *et al*. [23] reported increases in leptospirosis cases is relation to sports and recreational activities in nature, but this was not observed in our county.

Annual reports of the Croatian Institute for Public Health 1990–2007 do not show any epidemiologic changes over time, but the indication that leptospirosis became a more severe disease with higher mortality [24]. Unfortunately, there is no consensus for the definition of severity of leptospirosis. It is common that extremely severe cases undergo Weil’s syndrome. Some studies define severe pulmonary haemorrhagic syndrome (SPHS) as severe cases, others those who die during hospitalization [9, 25–29]. We evaluated severity of illness using the scores made by Kuzman during the great epidemic of haemorrhagic fever in Croatia in 2002 [15]. Our data show significant changes in the clinical manifestation of leptospirosis: during 1970–1975 severe clinical cases undergo only 10/113(8.8 %) patients [14], in our study (2000–2004) it was three times higher (7/24, 29.2 %; Table 3). Among our *Leptospira* culture-positive patients (and *Leptospira* identified species), 6/10(60.0 %) suffered a severe course of disease (Table 1), four of whom presented with Weil's syndrome and two with SPHS; no fatal outcome was recorded. The trend toward severe clinical presentations continued in the following years [16].

Special attention in this study was given to patients (no. 14) in whom *Leptospira* was isolated, and data saved until the present. Leptospirosis was mostly indicated by conjunctiva injection and severe muscle pains, less frequently by jaundice and renopathy (Table 4). The sedimentation rate and LDH were elevated in all patients with positive culture irrespective to the severity of the disease, whereas thrombocytopenia was significant for all but mild infection. Other increased laboratory markers correlated more strongly with severity of clinical presentation of leptospirosis (Table 5). Comparative results were found also in other studies [7, 8, 24, 30].

Seroepidemiological and seroepizootiological studies in Croatia based on the MAT test have shown changes in the incidence of probable infectious serogroup in both animals and humans. Studies show continuous presence of Grippotyphosa and Icterohaemorrhagiae serogroups, reduced frequency of Sejroe (serovar Saxkoebing), and increasing frequency of Australis serogroup [14, 16, 22, 30, 31]. In the period of our study, serogroup Icterohaemorrhagiae remained dominant (Table 1).

Given in mind that our work is based on human isolated *Leptospira* while former on the blood MAT results, it is difficult to compare results. It is known that cross-reactions frequently occur in the serum of infected patients, allowing the presumptive identification of the serogroup involved in the human disease [2, 12, 23].

In addition to Icterohaemorrhagiae, Grippotyphosa was also isolated in our area, although it was found far less commonly (Table 1). Interestingly, a new serogroup, either Batavia or Tarassovi emerged in our geographic area (Table 1); it could not be identified because agglutinated with both hyperimmune sera. Moreover, we had no strain with such a pattern of NotI-RFLP in our *Leptospira* collection. The strain belonged to *L. kirschneri *, different from all other isolated *Leptospira* strains that were found to be *L. interrogans sensu stricto* (with exception of serovar Grippotyphosa that we were not able to identify species, Table 1). Štritof-Majetić *et al*. [32] identified *Leptospira* strains isolated from the animal reservoir in the eastern part of Croatia; regarding to species level, 60 % of isolated strains belonged to *L. kirschneri *, 30 % to *L. interrogans sensu stricto* and 20 % to *L. borgpetersenii *, regarding to the serogroup 50 % belonged to serogroup Pomona, 30 % to Australis, and remaining 20 % to Sejroe, Batavia and Grippotyphosa [32]. The data indicate different affinity of particular species and/or serovar to particular host (human or animal) but also can reflect different distribution of strains in different geographical areas of Croatia. Moreover, Turk *et al*. [33] reported about human *Leptospira* strains isolated from Croatian inland patients; interesting, only half of these strains belonged to *L. interrogans *, serogroup Icterohaemorrhagiae that was our dominant isolate. Although we were able to perform MLST determination for only six strains, all our strains took place in two positions in minimum spanning tree (Fig. 3). Our strains were positioned close to other either human or animal isolates also including strain ST 144 of *L. kirschneri* emphasizing their transmission from animals to humans. Although MLST does not intend to replace serological determination of isolated strains, could be useful for epidemiological studies and genotype analysis.

Although many clinical features can support leptospirosis, microbiological confirmation is quite demanding. *Leptospira* isolation is a complex and long-lasting procedure, and despite its high specificity, it is rarely used in laboratory diagnosis, generally in reference laboratories [2, 12, 17, 34]. The low sensitivity of the method lies dominantly in a small number of bacteria in clinical material (previously antimicrobial therapy, antibody production, bacteria death due to improper or long lasting transport, other). The sampling itself is also important, and laboratory must ensure that clinicians have the necessary transport medium for immediate inoculation of patients' samples (which we did in patients from our study). Generally, culture requires a laboratory organization and collaboration between interested participants [2, 12, 17, 34]. All *Leptospira* strains isolated from our patients were isolated from the first clinical specimens, all from patient’s blood; isolation rate was 27.5 %(14/51). Data on low sensitivity of cultivation can also be found in published reports [12, 34].

In last two decades molecular methods (PCR) were implemented in diagnosis of leptospirosis trying to overcame fastidious culture procedure and time spent to achieve result [2, 12, 35]. In the time of our study, years 2000 to 2004, we were not able to perform PCR, later on we did not have samples any more.

Daily laboratory diagnosis of leptospirosis is based primarily on serology (e.g. MAT) that is demanding regarding to time and expertise of laboratory personnel [25]. MAT requires the collection, maintenance and monthly checking of *Leptospira* strains. Safety issues relating to working with live cultures are also important. Moreover, interpretation of MAT results are fastidious. If a patient is infected with a serovar not included in the agglutination test, the test result may be a false negative [2, 12, 36]. In the acute phase of the disease the sensitivity of the test is low; when antibodies appear, cross agglutination among different serological groups is frequent [2, 12, 37]. Moreover, MAT does not allow monitoring dynamics of IgM and/or IgG [12]. Nonetheless, the MAT test is the reference one for diagnosis of leptospirosis [2, 12].

Among the 51 patients who were tested for antibodies in our study, MAT confirmed infection in 11/51(21.5 %) compared with confirmation by cultivation in 14/51(27.5 %). The serum sampling before antibodies appeared could explain the majority of negative results. For the majority of our patients that were seronegative at first testing, we did not receive additional samples, partly because infection was confirmed by culture, and partly because leptospirosis was soon after excluded by clinicians since patients had undergone short-term fever that rapidly recovered. The importance of monitoring patients in the following months was also emphasized in the literature [38]. Limmathurotsakul *et al*. [39] also reported about the great number of serologically negative patients infected with *Leptospira *. He found that negative results could be because of high heterogeneity of lipopolysaccharide antigens that provoke different immune response and, in case serovar is not included in the test, the obtained result of the test MAT is negative. On the other hand, the main advantage of MAT is its high specificity. Although cross-reactivity with other serovars may occur in the beginning of infection, however, litres of non-specific antibodies rapidly fall down while antibodies specific to the serovar implementing in the infection remain in the blood for long time period, also for years [12].

Taking together, from Tables 2 and 3 is evident that clinically suspected leptospirosis has been confirmed by microbiological tests in most patients at their first examination; culture (no. 14) and MAT (no. 11) contributed to diagnosis almost equally, no statistically significant differences were found between them (*P*=0.453). Our patients could be an ideal group for comparing two microbiological approaches but, unfortunately, due to the distance and difficult cooperation between laboratory and hospital (two different countries), we could compare two tests in only 13 patients whose samples were taken simultaneously for culture and MAT. Although our results indicate correlation between isolation rate and absence of antibodies, the tested sample was too small for the definitive conclusion. Data about negative cultivation in serologically positive patients can be found in the literature [12, 40].

## Conclusion

The first report about *Leptospira* isolates from humans in Koprivnica-Križevci County reveal that most patients (80 %) were infected by *Leptospira* of the serological group Icterohaemorrhagiae, followed by Grippotyphosa. Regarding species, most strains belonged to *L. interrogans sensu stricto* (80 %), followed by *L. kirschneri *. Leptospirosis in period 2000–2004 in our region remained disease of the rural population, most commonly manifested as moderate severe clinical course, and can be equally diagnosed by serology and/or cultivation.
